# The DmsABC S-oxide reductase is an essential component of a novel, hypochlorite-inducible system of extracellular stress defense in *Haemophilus influenzae*

**DOI:** 10.3389/fmicb.2024.1359513

**Published:** 2024-04-04

**Authors:** Marufa Nasreen, Daniel Ellis, Jennifer Hosmer, Ama-Tawiah Essilfie, Emmanuelle Fantino, Peter Sly, Alastair G. McEwan, Ulrike Kappler

**Affiliations:** ^1^School of Chemistry and Molecular Biosciences, Australian Infectious Diseases Research Centre, The University of Queensland, St. Lucia, QLD, Australia; ^2^QIMR Berghofer Medical Research Institute, Herston, QLD, Australia; ^3^Child Health Research Centre, South Brisbane, QLD, Australia

**Keywords:** respiratory infection, *Haemophilus influenzae*, sulfoxide reduction, oxidative stress, host-pathogen interactions

## Abstract

Defenses against oxidative damage to cell components are essential for survival of bacterial pathogens during infection, and here we have uncovered that the DmsABC S-/N-oxide reductase is essential for virulence and in-host survival of the human-adapted pathogen, *Haemophilus influenzae*. In several different infection models, *H. influenzae* Δ*dmsA* strains showed reduced immunogenicity as well as lower levels of survival in contact with host cells. Expression of DmsABC was induced in the presence of hypochlorite and paraquat, closely linking this enzyme to defense against host-produced antimicrobials. In addition to methionine sulfoxide, DmsABC converted nicotinamide- and pyrimidine-N-oxide, precursors of NAD and pyrimidine for which *H. influenzae* is an auxotroph, at physiologically relevant concentrations, suggesting that these compounds could be natural substrates for DmsABC. Our data show that DmsABC forms part of a novel, periplasmic system for defense against host-induced S- and N-oxide stress that also comprises the functionally related MtsZ S-oxide reductase and the MsrAB peptide methionine sulfoxide reductase. All three enzymes are induced following exposure of the bacteria to hypochlorite. MsrAB is required for physical resistance to HOCl and protein repair. In contrast, DmsABC was required for intracellular colonization of host cells and, together with MtsZ, contributed to resistance to N-Chlorotaurine. Our work expands and redefines the physiological role of DmsABC and highlights the importance of different types of S-oxide reductases for bacterial virulence.

## Introduction

Sulfur-containing molecules such as glutathione, coenzyme A and the amino acids cysteine and methionine are essential for the maintenance of cellular redox balance, metabolic pathways, and enzyme function and regulation. However, the same high reactivity that is essential for their cellular function makes sulfur-containing molecules targets for inactivation by oxidation (Staerck et al., 2017; Gout, 2019; Kappler et al., 2019).

S-oxide reductases are enzymes that can reverse sulfoxide-formation on biomolecules, including proteins, sulfur-containing amino acids and vitamins such as methionine, cysteine and biotin that are highly susceptible to oxidative damage (Baltes et al., 2003; Denkel et al., 2013; Dhouib et al., 2016, 2021; Ezraty et al., 2017; Kappler et al., 2019; Zhong et al., 2020; Nasreen et al., 2021). S-oxide reductases are emerging as essential components for virulence and fitness of bacterial pathogens such as *Escherichia coli*, *Salmonella* sp., *Actinobacillus pleuropneumoniae*, and *Haemophilus influenzae* (Baltes et al., 2003; Denkel et al., 2013; Dhouib et al., 2016, 2021; Ezraty et al., 2017; Kappler et al., 2019; Zhong et al., 2020; Nasreen et al., 2021), where a loss of S-oxide reduction causes a reduction of survival in infection models.

*Haemophilus influenzae*, the focus of this study, is a human-adapted pathobiont that colonizes the nasopharynx as a commensal but causes acute diseases such as otitis media, sinusitis or pneumonia on access to the upper or lower respiratory tract (Van Eldere et al., 2014; Wen et al., 2020). Non-encapsulated, so-called ‘non-typeable’ strains of *H. influenzae* are currently the most common type of clinical isolate. These strains contribute to the pathogenesis and significantly worsen exacerbations of chronic lung diseases such as asthma, chronic obstructive pulmonary disease (COPD), cystic fibrosis and recovering COVID-19 patients (King, 2012; Van Eldere et al., 2014; Langereis and de Jonge, 2020; Lansbury et al., 2020; López-López et al., 2021; Short et al., 2021). Diseases caused by *H. influenzae* are characterized by high rates of recurrence and persistence (Ahearn et al., 2017), and sites of *H. influenzae* infections show high levels of inflammation and oxidative stress that lead to sulfoxide damage to proteins and redox molecules (Tufvesson et al., 2015; Saliu et al., 2021).

Sulfoxide formation can be caused by host-produced extracellular stressors such as hypochlorite or N-Chlorotaurine (Gottardi and Nagl, 2010; da Cruz Nizer et al., 2020). These extracellular chemical stressors first impact the bacterial cell envelope and periplasm, where we have recently described three enzymes that have the capacity to protect *H. influenzae* from sulfoxide stress. These include a strictly conserved periplasmic peptide methionine sulfoxide reductase, MsrAB, that repairs sulfoxide damage to proteins using thiol-based catalysis (Nasreen et al., 2020, 2022) and the two molybdenum-containing, periplasmic S- and N-oxide reductases, DmsABC and MtsZ (Dhouib et al., 2016, 2021; Struwe et al., 2021). Of these two enzymes, MtsZ is present in about 80% of strains and is strongly associated with methionine sulfoxide (MetSO) reductase activity, while DmsABC appeared to have a negligible contribution to this activity and had no identifiable *in vitro* phenotype, but is found in all *H. influenzae* strains (Othman et al., 2014; Dhouib et al., 2016). Despite this, in a mouse model of lung infection, attenuation of a *H. influenzae* Hi2019^Δ*dmsA*^ strain was much more significant than for the Hi2019^Δ*mtsZ*^ strain (Othman et al., 2014; Dhouib et al., 2016, 2021). These results suggest that *H. influenzae* DmsABC is required during conditions that exist only during contact with host cells. However, at present, it is not known what conditions, other than an anaerobic environment, lead to an induction of the operon that encodes DmsABC (Dhouib et al., 2021), and it is also unclear what substrate(s) DmsABC converts during infection. Equally unclear is whether there is a functional connection between the three S-oxide reductases in *H. influenzae*, and specifically DmsABC and MtsZ.

To address these gaps in knowledge, we have investigated the role of DmsABC for virulence of *H. influenzae* strains carrying either both DmsABC and MtsZ or only DmsABC in different infection models, including changes in the host response to infection. We were able to link expression of *dmsABCDE* to the presence of hypochlorite, a reactive chlorine species produced by the human immune system during infection, and identified several small molecule S- and N-oxides as potential natural substrates for DmsABC.

Together with the MsrAB peptide methionine sulfoxide reductase, DmsABC and the functionally related enzymes, MtsZ, form a novel, hypochlorite-inducible extracellular stress defense system in *H. influenzae*. This system protects *H. influenzae* from reactive chlorine and oxygen species and is essential for survival of the bacteria during infection.

## Materials and methods

### Bacterial strains and growth conditions

*E. coli* strains (Supplementary Table S1) were grown on liquid or solid Luria−Bertani (LB) medium at 37°C for 16–18 h with shaking at 200 rpm for liquid media. *Haemophilus influenzae* (Hi) 2019 (Campagnari et al., 1987) (NCBI acc. no.: NZ_CP008740.1), NTHi 86-028NP (Bakaletz et al., 1989) (NCBI acc. no.: NC_007146.2) and derivative strains (Supplementary Table S1) were grown on Brain Heart Infusion broth (BHI, BBL) or agar supplemented with hemin (10 μg/mL) and NAD (10 μg/mL) at 37°C for 16 h and 200 rpm shaking for broth cultures. Chemically Defined Medium (CDM) was also used and contained 10 mM glucose, 1 mM sodium pyruvate, 0.08 mg/mL uracil, 0.17 mg/mL inosine, 10 μg/mL β-NAD, 25 mM HEPES, pH 7.4, 10 μg/mL hemin and 2 mg/mL NaHCO_3_ in RPMI1640 (Coleman et al., 2003). The sBHI and CDM media were also used to cultivate *H. influenzae* clinical isolate strains from our collection. *Actinobacillus pleuropneumoniae* (strain 4074) (Supplementary Table S1) was cultivated on CDM medium under microaerobic conditions at 37°C and 45°C, respectively. Ampicillin (100 μg/mL *E. coli*), kanamycin (100 μg/mL *E. coli*; 10 μg/mL Hi) and tetracycline (10 μg/mL *E. coli*, 1 μg/mL Hi) were added to culture media when needed.

NTHi growth experiments (Hosmer et al., 2022) were conducted in microtiter plates, each well containing 200 μL of CDM medium. Three biological replicates per strain were incubated at 37°C and 200 rpm using a Clariostar multimode plate reader (BMG LabTech). The atmospheric control unit was set up to allow approximately 20% O_2_ for aerobic conditions, 2.8% O_2_ for microaerobic conditions, and 5% CO_2_ with no oxygen for anaerobic conditions (no shaking). Growth rates were calculated according to the method described in Kurokawa and Ying (2017).

### Transformation of *Haemophilus influenzae* strains

NTHi transformation was performed as in Poje and Redfield (2003). Briefly, *H. influenzae* cultures (20 mL) were grown to an OD_600nm_ of 0.25 using sBHI, harvested, and washed twice in freshly prepared MIV solution (Poje and Redfield, 2003) before resuspension in 5 mL MIV and incubation at 37°C with shaking for 100 min to develop competence. One microgram of linearized plasmid was added to 1 mL of competent cells and incubated at 37°C for 30 min before 1 mL of sBHI was added. Samples were incubated at 37°C for 1 h before plating on sBHI plates with 20 μg/mL kanamycin. MIV solution was prepared by combining 25 mL solution 21 (per liter: 4 g L-aspartate, 0.2 g L-glutamate, 1 g fumarate, 4.7 g NaCl, 0.87 g K_2_HPO_4_, 0.76 g KH_2_PO_4_, 0.2 mL Tween 80, pH 7.0) with 0.25 mL solution 22 (4 mg L-cytosine, 10 mg L-tyrosine dissolved in 1 mL 1 M HCl at 37°C followed by addition of 9 mL H_2_O and 6 mg L-citrulline, 20 mg L-phenylalanine, 30 mg L-serine, 20 mg L-alanine), 0.25 mL 0.1 M CaCl_2_, 0.25 mL 0.05 M MgCl_2_, and 0.25 mL 5% Difco vitamin-free Casamino acids.

### Biofilm formation

Biofilm formation in 96-well microtiter plates used the protocol described in Schembri and Klemm (2001), and bacterial survival in biofilms was determined as in Dhouib et al. (2016) using proteinase K digestion and serial dilution. Briefly, non-typeable *H. influenzae* strains were grown to OD_600nm_ of 0.2–0.3 under microaerobic conditions in sBHI broth at 37°C with shaking. Then, the cultures were diluted to an OD_600nm_ of 0.05 and dispensed into 96-well microtiter plates (100 μL per well, 96-well plate: Techno Plas, cat no: SMPSL) and incubated at 37°C. Following incubation, planktonic cells were removed by washing the wells twice with sterile water, and the wells were subsequently stained using 0.1% crystal violet for 10 min. After plate drying, 30% acetic acid was applied to dissolve the biofilm, and the optical density at 550 nm (OD_550nm_) was measured using a plate reader. To determine viable cell counts, the wells of the biofilm plate were rinsed twice with sterile water, followed by incubation with 100 μL of 0.1 mg/mL protease K for 10 min at room temperature to dissolve the biofilm. Subsequently, samples were collected, serially diluted in 1xPhosphate Buffered Saline (PBS), and plated on sBHI agar to quantify the colony forming units (CFU) per well. Each strain was analyzed with three biological replicates, and several technical replicates were used for each biological replicate.

### Oxidative and nitrosative stress susceptibility testing

Bactericidal assays were conducted following the protocol described in Nasreen et al. (2020) and used 150 and 175 μM HOCl, 0.5–1.5 mM NCT. In all experiments, HOCl was applied as NaOCl. *H. influenzae* 2019 strains, obtained from freshly grown sBHI plates, were resuspended to an OD_600nm_ of 1.0 (∼6 × 10^8^ CFU/mL) in 1xPBS. Subsequently, 900 μL of bacterial culture were combined with 100 μL of a freshly prepared 10x stock of the test compound or sterile water as a control. The reactions were incubated at room temperature for 60 min with orbital shaking, followed by immediate serial dilution and plating on sBHI plates for CFU/mL determination.

For S-oxide reductase activity assays in *H. influenzae* strains 2019 and 86-028NP, cultures growing microaerobically in CDM were treated with 0.4 mM HOCl or 10 mM paraquat for 60 min at mid-exponential phase before harvesting.

Nitrosative stress susceptibility was assessed following the method of (27). NTHi strains, with three biological replicates grown on sBHI agar plates, were inoculated in 50 mL sBHI broth in 50 mL tubes and incubated at 37°C in a CO_2_ incubator for 16–18 h. These cultures were then used to inoculate sBHI broth buffered to pH 5.5 at an initial OD_600 nm_ of 0.07. 150 μL of this bacterial suspension were added to each well of a round-bottom 96-well microtiter plate (Costar 3799), followed by addition of 150 μL of 20 mM or 30 mM NaNO_2_ to each reaction to achieve final concentrations of 10 mM and 15 mM. Sterile water was added to control wells. Plates were incubated at 37°C, 5% CO_2_, for 16 h in an anaerobic jar using Anaerocult A catalysts (Merck) to remove oxygen. After 2 h of incubation, the cultures were serially diluted and plated on sBHI plates for CFU/mL determination. Growth rate determinations used the program GrowthRates v.6.2.1 (Hall et al., 2013).

### General molecular biology and biochemical methods

Standard methods were used throughout (Ausubel et al., 2005). All chemicals were purchased in analytical grade or equivalent. Genomic DNA was isolated using DNAzol reagent (Thermo Fisher Scientific). GoTaq Green MasterMix (Promega) was used for general PCR (primer sequences, Supplementary Table S2), the GeneJET Plasmid Miniprep Kit and GeneJET PCR Purification kit (both Thermo Fisher Scientific) for plasmid and PCR product purification. Restriction enzymes were from Thermo Fisher Scientific, T4 ligase (NEB) was used for ligations. Cell-free extracts of NTHi were generated using BugBuster Mastermix (Novagen) according to the manufacturer’s instructions (750 μL were used for pellets from 50 mL culture). Protein concentrations were determined using the BCA-1 kit (Sigma Aldrich).

### Construction of *Haemophilus influenzae* 86-028*^ΔdmsA^*

The previously constructed plasmid, pGEM-Hi*dmsA::kan* (Dhouib et al., 2021) was linearized using *NcoI* and transformed into competent NTHi 86-028NP using the method described above.

### Construction of double mutants of Hi2019

The *tet* resistance cassette, amplified from pRK415 using primers from Supplementary Table S2, was ligated into two previously constructed plasmids: pGEM-Hi*mtsZ* (Dhouib et al., 2016) and pBlu-Hi*msrAB* (Nasreen et al., 2020). This resulted in the construction of pGEM-Hi*mtsZ::tet* and pBlu-Hi*msrAB::tet* plasmids. The linearized pGEM-Hi*mtsZ::tet* plasmid was transformed into Hi2019^Δ*dmsA*^ *and* Hi2019*^ΔmsrAB^* to generate Hi2019*^ΔdmsAΔmtsZ^ and* Hi2019*^ΔmsrABΔmtsZ^.* Additionally, linearized pBlu-Hi*msrAB::tet* was used to create double mutant Hi2019*^ΔdmsAΔmsrAB^.*

### RNA isolation and cDNA synthesis

Samples for RNA isolation from bacteria were obtained during the mid-exponential growth phase (OD_600nm_ Hi, Ap ∼ 0.45) from cultures growing on CDM under microaerobic conditions. Additionally, RNA samples were collected at 30, 60, and 120 min after treating cultures with 200 μM HOCl, 150 μM H_2_O_2_, or 5 mM paraquat, following the method outlined in reference (Pauwels et al., 2004).

For each sample, 2 mL of culture was preserved using 2 mL of RNAprotect Bacteria Reagent (Qiagen) before RNA isolation using the Illustra RNAspin Mini RNA isolation kit (Cytiva). RNA from snap-frozen mouse lung tissue was isolated by homogenization in 1 mL Trizol (Thermo Fisher Scientific), followed by centrifugation to remove large cellular debris and RNA isolation according to the manufacturer’s instructions. Genomic DNA was removed using the Turbo DNAfree kit (Thermo Fisher Scientific), and RNA concentrations were determined using the Quant-it RNA HS kit (Thermo Fisher Scientific). The absence of genomic DNA was confirmed by PCR using 16S primers and β-actin (*ACTB*, mouse) primers (Supplementary Table S2), with purified RNA serving as the template. Subsequently, cDNA was synthesized from 500 ng of genomic DNA-free RNA using random hexamer primers and either the Superscript IV VILO Master Mix (Thermo Fisher Scientific) or the Lunascript RT SuperMix kit (New England Biolabs), following the manufacturer’s instructions.

### Quantitative RT-PCR

Quantitative reverse transcription-PCR (qRT-PCR) was conducted following the procedures outlined in reference (Othman et al., 2014). Each qRT-PCR reaction (10 μL) comprised diluted cDNA (1:100) as the template, QuantiNova SYBR Green qPCR Master Mix from Qiagen, and specific oligonucleotide primers (Conc 2 μM) listed in Supplementary Table S2. Reference genes used included gyrase (*gyrA*) genes for *H. influenzae, Actinobacillus pleuropneumoniae,* and β-actin (*ACTB*) for mouse cDNA. Data analysis and normalization were performed as in Kappler et al. (2005). The cycle threshold (CT) value for all samples was determined using QuantstudioTM Real-time PCR software version 1.3 (Thermo Fisher Scientific). The PCR efficiencies were determined using LinRegPCR software version 2016.0 (Ruijter et al., 2009).

### S-/N-oxide reductase activity assays

S- and N-oxide reductase activity assays were performed anaerobically in 20 mM sodium phosphate buffer (pH 6.8) containing 0.2 mM Methyl viologen, 0.3 mM sodium dithionite and one of the following terminal electron acceptors: 10 mM Dimethyl Sulfoxide (DMSO), 10 mM L-Methionine Sulfoxide (L-MetSO), 10 mM S-Biotin Sulfoxide (S-BSO), 10 mM racemic methyl p-tolyl sulfoxide (MPTS), 10 mM R-MPTS & S-MPTS, 2 mM Pyrimidine N-Oxide (Pyr N-Ox), and 5 mM Nicotinamide N-Oxide (Nic N-Ox). The re-oxidation of reduced methyl viologen [ɛ_600_ = 13.7 mM^−1^ cm^−1^ (Watanabe and Honda, 1982)] was monitored at 37°C in a Cary60 Spectrophotometer (Agilent Technologies) as described in Dickie and Weiner (1979). For determination of kinetic parameters, the concentrations of the substrates were varied. Specific enzyme activities are given as μmoles of substrate reduced per min (U) and mg of protein present. Kinetic data were fitted using Prism 9 (GraphPad). For enzyme assays using purified *H. influenzae* MtsZ, the protein was prepared as described in Dhouib et al. (2016) and Struwe et al. (2021).

### NTHi host cell adherence and invasion assays using cultured tissue cells and normal human nasal epithelia

The Human Bronchial Epithelial 16HBE14 cell line was cultured using Minimal Essential Medium (MEM) supplemented with 10% heat-inactivated Fetal Bovine Serum (FBS). Adherence and invasion assays for NTHi were conducted following the procedures outlined in Dhouib et al. (2015). Normal Human Nasal Epithelia (NHNE) were prepared following the protocol outlined in Yeo et al. (2019) using primary nasal cells obtained from healthy donors at the Child Health Research Centre, UQ, Brisbane. Seven days before infection, NHNE were transferred to steroid- and antibiotic-free PneumaCult-ALI medium (Stemcell). NTHi for infection were resuspended in 1xPBS and applied at a Multiplicity of Infection (MOI) of 10:1. Uninfected NHNE were exposed to an equal amount of sterile 1xPBS. Following a 24-h infection period at 37°C with 5% CO_2_, the inoculum was removed by washing with 1xPBS, and the basal medium was replaced.

Post-infection, NHNE apical surfaces were washed every second day using 100 μL 1xPBS, and the basal medium was replaced. Lysates were generated by a 10 min incubation of NHNE with 1% saponin at 37°C. Intracellular bacterial Colony Forming Units (CFU) were determined after treating the NHNE surface with gentamycin (100 μg/mL) for 1 h at 37°C. Gentamycin was then aspirated, and the apical compartments were washed five times with 200 μL of 1xPBS before lysing the NHNE with a 10 min incubation at 37°C with 1% saponin. Bacterial loads in lysates were determined by plating serial dilutions on sBHI agar.

NHNE transepithelial electrical resistance (TEER) was measured every 2 days using a Millicell ERS-2 Volt-ohm meter. For competition assays, NHNE were infected with an equal mixture of Hi2019^WT^ and one mutant strain (total MOI 10:1), and bacterial loads were analyzed as described above. However, serial dilutions were plated on sBHI to determine total bacterial numbers and on sBHI with 20 μg/mL kanamycin to assess the load of NTHi carrying a mutation. Hi2019^WT^ cell numbers were determined by subtracting CFU/mL of kanamycin-resistant (mutant) NTHi from total NTHi CFU/mL for the same time point. Each infection and co-infection involved two biological replicates for each strain or strain combination, with three technical replicates used for CFU/mL determination in each biological replicate.

### Mouse bone marrow macrophage infection assay

The procedure for isolating Bone Marrow Macrophages (BMM) from C57 Black mice was adapted from Tushinski et al. (1982). In summary, mice were sacrificed, and femurs and tibias were collected, with muscles and surrounding tissues removed. The tips of the femurs and tibias were excised using surgical scissors, and the bone cavity was flushed with complete macrophage medium [RPMI1640 containing 10% heat-inactivated FBS 2 mM L-glutamine (GlutaMAX, Thermo Fisher Scientific) and 150 ng/mL recombinant colony-stimulating factor-1 (rCSF-1, produced at Protein Expression Facility, The University of Queensland)].

Bone marrow cells were washed twice with complete medium (centrifuged at 300 × g for 5 min each time) before being evenly distributed into eight sterile, 100 mm square petri dishes, each containing 15 mL of complete medium. Penicillin/streptomycin (Stock: Penicillin: 10,000 Units/mL, Streptomycin: 10,000 mg/mL) was added to the media to a final concentration of 1%, and the plates were incubated for 6 days at 37°C with 5% CO_2_. On day 3, fresh rCSF-1 (final conc.: 150 ng/mL) was added. On day 6, the growth medium was discarded, and BMMs were washed off the plate using 10 mL of 1xPBS. BMMs were then centrifuged at 300 × g for 5 min and resuspended in antibiotic-free BMM medium. Subsequently, 1 × 10^5^ cells/well were seeded into 24-well tissue culture plates for the infection assay.

The infection assays used a MOI of 1,000:1. After 1 h, the inoculum was removed, and complete macrophage medium with 7 μg/mL polymyxin B was added to the wells for 60 min to eliminate extracellular bacteria. For 2 h infections, cell layers were washed twice with RPMI before 100 μL of 0.1% saponin was used to lyse the macrophages. Intracellular bacteria were quantified through dilution plating. For 4 and 6 h infections, the medium containing 7 μg/mL polymyxin B was replaced with medium containing 0.5 μg/mL polymyxin B for 2 h and 4 h before processing the cells to determine intracellular bacteria.

### *Haemophilus influenzae* murine lung infection

For NTHi pulmonary infection, the mouse model of lung infection we described previously was used (Morey et al., 2013; Dhouib et al., 2016). Hi2019 and Hi2019*^ΔdmsA^* were cultured on sBHI plates for 16 h at 37°C with 5% CO_2_. A bacterial suspension in 1xPBS, containing 10^7^ CFUs in 30 μL, was intranasally inoculated into female BALB/c mice aged 5 to 6 weeks. Groups of 5 mice were euthanized and necropsied at 0 h, 6 h, 24 h, 48 h, and 72 h, as described in Hosmer et al. (2022). Lungs were aseptically removed from sacrificed mice, and lung tissue was either snap-frozen in liquid nitrogen for RNA isolation or homogenized in 1 mL 1xPBS. The homogenized samples were serially diluted and plated on sBHI agar to determine CFU/mL. Additionally, bronchoalveolar lavage fluid (BALF) was collected, and CFU/mL were determined. CFUs per lung were calculated as described in Essilfie et al. (2012). Giemsa staining of BALF was performed according to the manufacturer’s instructions (Sigma-Aldrich). Statistical comparisons of mean CFU/lung and BALF were carried out using two-tailed *t*-tests integrated into the Prism 9 software package.

### ELISA assays

Mouse BALF was used for ELISA assays (Thermo Fisher Scientific) according to the manufacturer’s instructions to detect the presence of mouse TNFα and IL-6. Data were collected for all mice in each treatment group. The statistical significance of the detected differences among the groups was assessed using Prism 9 (GraphPad).

### Statistical analyses

Statistical analyses were carried out using Prism versions 9 and 10 (GraphPad). Statistical comparisons of mean CFU/lung and BALF following mouse lung infection experiments were carried out using two-tailed *t*-tests. All other analyses used either 1-Way of 2-Way ANOVA with an appropriate multi-comparison correction. All analyses used a *p* < 0.05 to determine statistical significance. Details of the analyses are provided in the Figure legends.

### Ethics statement

Experimental animal procedures were conducted in strict accordance with the recommendations outlined in the QLD Animal Care and Protection Act (2001) and the Australian Code of Practice for the Care and Use of Animals for Scientific Purposes, 8th edition. The protocols were approved by the Animal Care and Ethics Committees of QIMR Berghofer and the University of Queensland (QIMR/050/19).

Human nasal cell samples were obtained from healthy donors at the Child Health Research Centre, UQ, Brisbane, under the approval of the University of Queensland Ethics Committee (Approval #2017000520). Clinical isolates of *Haemophilus influenzae* were collected from routine pathology samples according to approvals 2021/HE001644, 2014/HE000067.

## Results

### Infection with an *Haemophilus influenzae* Δ*dmsA* strain elicits a reduced host immune response to infection in a mouse model of lung infection

**Figure 1:**
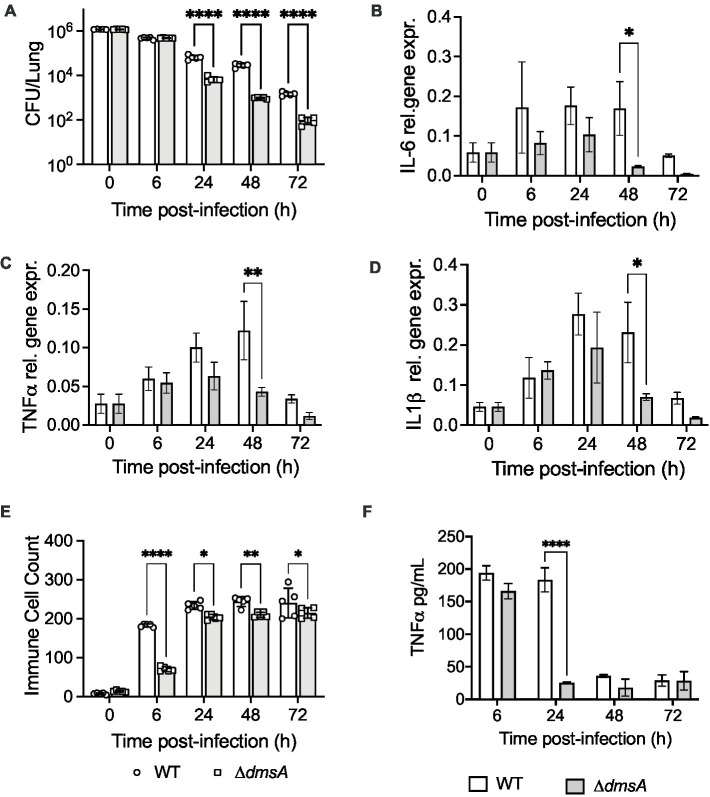
Comparison of mouse lung infections with Hi2019^WT^ and Hi2019^Δ*dmsA*^. **(A)** Bacterial colony forming unit (CFU) recovered per lung. **(B–D)** Relative expression of IL-6, TNFα and Il1β in mouse lung tissue during infection with NTHi strains. qPCR data was normalized against expression of ACTB, cDNA was generated using random hexamers. **(E)** Immune cell counts (Giemsa stain) in mouse Bronchoalveolar Lavage Fluid (BALF). **(F)** TNFα levels in mouse BALF determined by ELISA. Infection assays used five mice per group, qPCR analyses used RNA isolated from three mice for each data point. Statistical analyses: **(A)** multiple un-paired *t*-tests, ****p < 0.0001; **(B–F)** 2-Way ANOVA, Sidaks’ multiple comparison correction, *p < 0.05, **p < 0.01, ****p < 0.0001.

We have previously reported that *H. influenzae* DmsABC is required for virulence in a mouse model of infection (Dhouib et al., 2021), and to better understand this process, we investigated whether the presence or absence of DmsABC affected the host response to *H. influenzae* infection. Using a mouse model of lung infection (Dhouib et al., 2021), the Hi2019^Δ*dmsA*^ strain was cleared significantly faster than the wildtype, with first differences becoming apparent at 24 h post-infection (p.i.) (Figure 1A). Compared to the wildtype, the total lung-associated populations of Hi2019^Δ*dmsA*^ were reduced 10x, 28x, and 18x times at 24 h, 48 h, and 72 h post-infection (p.i.) (total CFU/lung: *p* < 0.0001). Attenuation was similar for bacteria detected in bronchoalveolar lavage fluid (BALF) (Supplementary Figure S1A). We then tested the host immune response to infection using qPCR for RNA isolated from lung tissue and ELISA for BALF samples. The faster clearance of Hi2019^Δ*dmsA*^ was associated with reduced expression of the pro-inflammatory markers IL6, TNFα and IL1β in mouse lung tissue, with expression levels returning to near baseline by 48 h p.i. In contrast, for infections with the wildtype strain, expression of these genes was still elevated at 72 h p.i. (Figures 1B–D). Similar expression patterns were also observed for Hif1α, TGFβ and the BIRC3 gene that encodes an anti-apoptotic effector and has shown increased expression in human tissue cells infected with wildtype *H. influenzae* (Nasreen et al., 2020) (Supplementary Figures S1B–D). In mouse BALF, a reduced immune response was also apparent for TNFα, but not IL6 levels (Figure 1F; Supplementary Figure S1E). These data suggested a reduced production of chemo-attractants during infection with Hi2019^Δ*dmsA*^ that was accompanied by a reduced influx of immune cells into the lungs of infected mice (Figure 1E; Supplementary Figures S1F–H). The difference was most prominent at 6 h p.i., but persisted up to 72 h p.i. and was primarily due to lower neutrophil numbers. Together with the reduced recovery of Hi2019^Δ*dmsA*^ from mouse lungs, this could indicate reduced survival of the Hi2019^Δ*dmsA*^ strain in contact with resident lung immune cells.

To investigate this possibility, we tested whether Hi2019^Δ*dmsA*^ showed increased susceptibility to killing by bone marrow-derived mouse macrophages. While we had previously demonstrated slightly increased susceptibility of Hi2019^Δ*dmsA*^ to neutrophil-mediated killing (Dhouib et al., 2021), in macrophage killing assays, this strain showed a statistically significant increase in intracellular survival compared to the wild type at 6 h p.i. (Supplementary Figure S2). As neutrophil numbers were low in Hi2019^Δ*dmsA*^ infection BALF samples, we conclude that the increased clearance of Hi2019^Δ*dmsA*^ in the mouse lung was not due to a greater sensitivity to killing by phagocytic cells such as tissue-resident macrophages.

### DmsABC is required for survival of *Haemophilus influenzae* in contact with primary human epithelia and transepithelial migration

The human respiratory tract is the only known natural niche of *H. influenzae* (Van Eldere et al., 2014), and as a result, *H. influenzae* infections in healthy mice are not stable and of short duration (Figure 1A). To determine whether the *dmsA* mutation also reduces *H. influenzae* survival in contact with human respiratory epithelia, we infected normal human nasal epithelia (NHNE) with either the Hi2019^WT^ or the Hi2019^Δ*dmsA*^ strain. NHNE are pseudostratified epithelia that are derived from primary human nasal cells (Pezzulo et al., 2011; Hosmer et al., 2022). NHNE are differentiated at the air-liquid interface and closely resemble primary human respiratory epithelia (Pezzulo et al., 2011; Hosmer et al., 2022).

**Figure 2:**
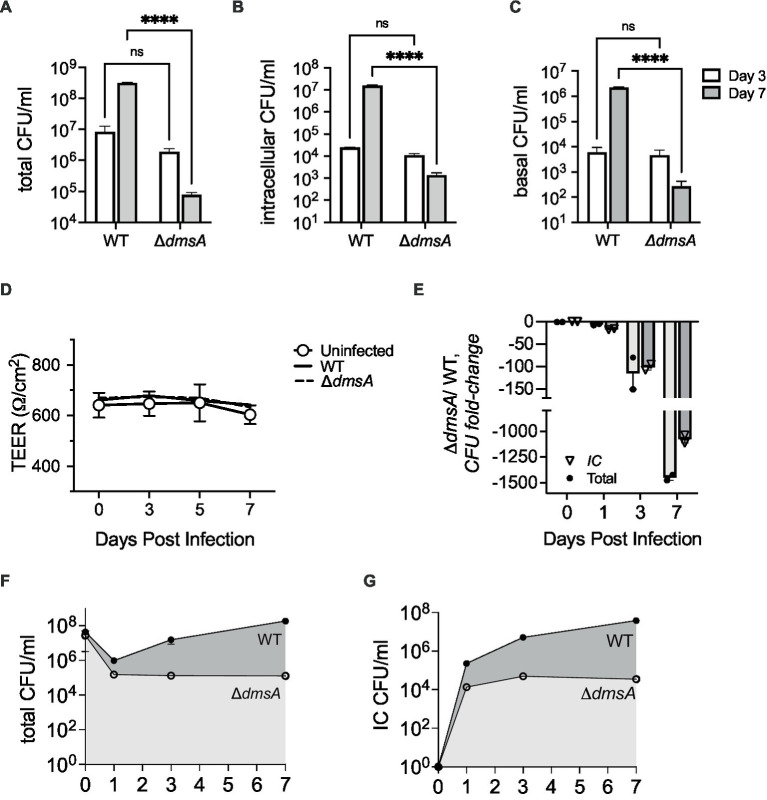
Infection of Normal Human Nasal Epithelia (NHNE) with Hi2019^WT^ and Hi2019^Δ*dmsA*^. **(A,B)** Total and intracellular bacterial colony forming unit (CFU) recovered from infected NHNE with a single NTHi strain. **(C)** Bacterial CFU/mL recovered from NHNE basal medium during single strain infections. **(D)** NHNE integrity during single strain infections determined using Transepithelial Resistance (TEER) values. **(E)** Changes in strain abundance (fold-change) during competitive infections of NHNE using a 1:1 mixture of Hi2019^WT^ and Hi2019^Δ*dmsA*^. IC, intracellular. **(F,G)** Total and intracellular CFU/mL recovered during competitive infections of NHNE using a 1:1 mixture of Hi2019^WT^ and Hi2019^Δ*dmsA*^. IC, intracellular. NHNE infections used *n* = 2 biological replicates, 3 technical replicates were used per biological replicate. Statistical analyses: 2-Way ANOVA, Sidaks multiple comparison correction, ****p < 0.0001. For panels **(F,G)**, the day 7 and day 3 and 7 values are significant with *p* < 0.0001. All other timepoint comparisons are not significant.

In NHNE single-strain infections, compared to the wildtype, attenuation of Hi2019^Δ*dmsA*^ increased from 3.6- and 2.2 -fold for total and intracellular bacteria on day 3 p.i. to 3 × 10^3^- and 1 × 10^4^- fold, respectively, by day 7 p.i. (Figures 2A,B). Transepithelial migration of Hi2019^Δ*dmsA*^ was also reduced, with only ~0.3% of total bacteria recovered for the mutant strain on day 7 p.i., compared to ~1.1% for the wildtype strain (Supplementary Figure S2C).

We then tested the survival of Hi2019^Δ*dmsA*^ in competitive infections of NHNE. Using an equal mixture of Hi2019^WT^ and Hi2019^Δ*dmsA*^, attenuation of the mutant strain was ~100 fold by day 3 p.i., and this increased to ~1,000 fold by day 7 p.i. for both total and intracellular bacterial populations (Figures 2E–G). Neither infection type appeared to cause significant damage to NHNE, as indicated by the consistency of TEER values between infected and uninfected control NHNE (Figure 2D; Supplementary Figure S3). These data confirmed that DmsABC is also required for the survival and virulence of *H. influenzae* in human nasal epithelia, its primary niche.

### Expression of Hi dmsABC is triggered by hypochlorite and other host-produced stressors

**Figure 3:**
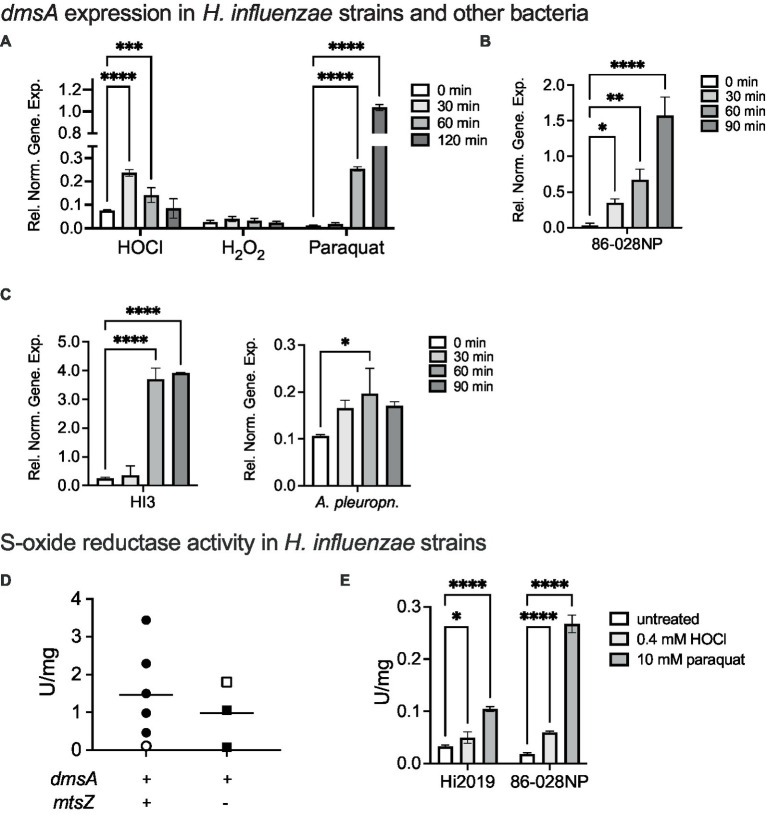
Induction of *dmsABC* gene expression and S-oxide reductase activity following exposure of bacteria to oxidative stressors. **(A)** Changes in *dmsABC* gene expression in Hi2019 following exposure of actively growing, microaerobic cultures to different oxidative stress challenges (200 μM HOCl, 150 μM H_2_O_2_, 5 mM paraquat). **(B,C)** Changes in *dmsABC* gene expression in *H. influenzae* strains 86-028NP and HI3 and *Actinobacillus pleuropneumoniae* 4,074 following exposure of actively growing, microaerobic cultures to 200 μM HOCl over a 90 min time course. **(D)** S-oxide reductase activity in *H. influenzae* strains with either a *dmsA*^+^/*mtsZ*^−^, or a *dmsA*^+^/− genotype following anaerobic growth. Open symbols denote the two control strains, Hi2019 and 86-028NP, respectively. **(E)** S-oxide reductase activity in actively growing, microaerobic cultures of *H. influenzae* strains 2019 and 86-028 following exposure to either 0.4 mM HOCl or 10 mM paraquat for 60 min. qPCR data and enzyme assays shown are averages of three determinations. Statistical testing used 2-Way-ANOVA with Dunnett’s multi-comparison correction for panels **(A,E)**, and 1-Way ANOVA (Holm-Sidak correction) for all other panels. *p < 0.05, **p < 0.01, ***p < 0.001, ****p < 0.0001. Only statistically significant comparisons are shown. In panel **(C)**, *A. pleuropneumoniae,* the 30 min and 90 min comparisons had *p*-values of 0.0509, i.e., were close to being significant.

Together with our previous investigations that documented the absence of an *in vitro* phenotype of Hi2019^Δ*dmsA*^ (Dhouib et al., 2021), the data above confirm that DmsABC is required under conditions that only exist when *H. influenzae* is in contact with host cells. This then suggested that host-produced stressors might play a role in controlling expression of *dmsABC*. To investigate this, we monitored expression of *dmsA* following exposure of actively growing, microaerobic cultures of Hi2019 to hypochlorite, hydrogen peroxide and or paraquat, a superoxide-producing agent. Hypochlorite caused a 3.3-fold increase in *dmsA* expression within 30 min of exposure, followed by a slow decrease in gene expression, while hydrogen peroxide had no or very little effect on *dmsA* expression (Figure 3A). Interestingly, the highest levels of *dmsA* expression were observed following paraquat exposure; however, induction of gene expression only occurred 60 min post-exposure (Figure 3A).

This is the first time that a specific, fast-acting inducer for *dmsA* expression other than general anaerobiosis has been identified in any bacterium, and we therefore tested whether hypochlorite induction of *dmsA* expression also occurs in other *H. influenzae* strains and other bacteria in which *dmsABC* genes are present such as *Actinobacillus pleuropneumoniae*. *A. pleuropneumoniae* is another bacterial pathogen of the Pasteurellaceae family for which a link between virulence and the presence of DmsABC has been previously reported (Baltes et al., 2003) (Figures 3B,C) Hypochlorite induced *dmsA* expression in all bacteria tested, but expression patterns and maximal induction levels varied, especially for the three *H. influenzae* strains. In most cases, increases in *dmsA* expression were measurable after 30 min, and peak expression occurred between 60 and 90 min post HOCl exposure. The smallest relative change, ~2-fold induction at 60 min, was observed for *A. pleuropneumoniae* (Figure 3C). The data clearly establish hypochlorite-induction as a feature of *dmsABC* regulation, suggesting a general role of DmsABC in bacterial stress responses during host-bacteria interactions in *H. influenzae* and other *Pasteurellaceae* (Baltes et al., 2003; Dhouib et al., 2021).

### DmsABC activity levels in *Haemophilus influenzae* show high strain-to-strain variation but did not correlate with the presence or absence of *mtsZ* in addition to *dmsABC*

As *H. influenzae dmsA* gene expression levels were very strikingly different between the three strains tested above, we hypothesized that this might correlate with the presence or absence of genes encoding the MtsZ MetSO reductase that is another S-oxide reductase in *H. influenzae* but is not strictly conserved. Accordingly, we compared S-oxide reductase activity levels in five genotypically *dmsABC*^+^/ *mtsYZ*^+^ strains and two *dmsABC*^+^/ *mtsZ*^−^ strains from our *H. influenzae* isolate strain collection. Hi2019 and Hi 86-028NP were used as reference strains for the two groups, respectively, and had 0.11 and 1.8 U/mg S-oxide reductase activity, respectively. Following anaerobic growth, total S-oxide reductase activities between 0.09 and 1.8 U/mg for the DmsABC-only group, and 0.11–3.44 U/mg for the DmsABC/MtsZ positive group of strains were determined (Figure 3D). Together, this indicates that under standard anaerobic conditions, S-oxide reductase activity varied significantly in a strain- but not genotype-dependent manner. It also shows that high S-oxide reductase activity is not a specific trait of recent clinical isolate strains.

To confirm that the observed induction of gene expression in the presence of HOCl and paraquat leads to an increase in S-oxide reductase activity, we then exposed the two reference strains to HOCl or paraquat prior to S-oxide reductase activity determination. This experiment confirmed induction of activity by 1.5- and 3.2-fold for Hi2019 and 86-028NP following HOCl exposure and 3.2- and 14.4-fold, respectively, for paraquat exposure (Figure 3E).

### Loss of DmsABC in an MtsZ-negative *Haemophilus influenzae* strain exacerbates the sensitivity to oxidative stress and loss of virulence

While the *dmsABC*^+^/ *mtsZ*^−^-genotype is only present in about 20% of *H. influenzae* isolate strains, these strains offer a unique opportunity to better understand the physiological role of DmsABC in *H. influenzae* pathogenesis and we therefore created and characterized a Hi86-028NP Δ*dmsA* strain.

**Figure 4:**
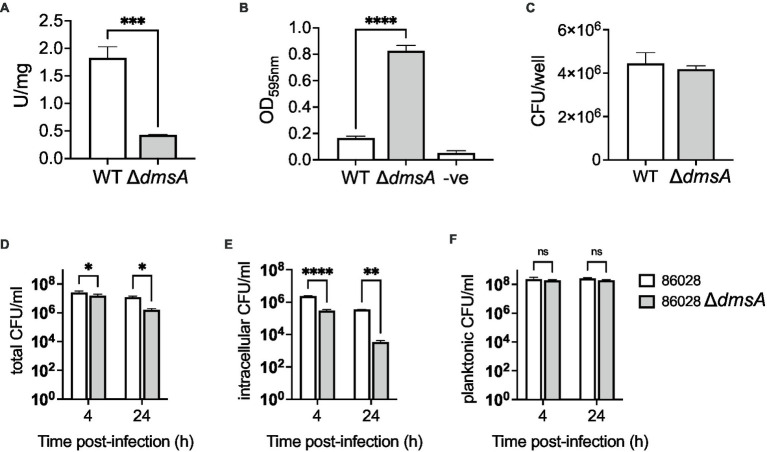
Properties of *H. influenzae* 86-028NP^WT^ and 86-028NP^Δ*dmsA*^ strains. **(A)** S-oxide reductase activity in *H. influenzae* 86-028NP^WT^ and 86-028NP^Δ*dmsA*^. **(B)** Biofilm formation in *H. influenzae* 86-028NP^WT^ and 86-028NP^Δ*dmsA*^ in 96-well plates under microaerobic conditions. Control wells (−ve) only contained sterile growth medium. Biofilms were stained using crystal violet. **(C)** Bacterial CFU/mL recovered from *H. influenzae* 86-028NP^WT^ and 86-028NP^Δ*dmsA*^ biofilms using protein K digestion. **(D–F)** Total, intracellular and planktonic CFU/mL recovered during infection of 16HBE14 human bronchial epithelial cells with either *H. influenzae* 86-028NP^WT^ or 86-028NP^Δ*dmsA*^ for 4 h or 24 h. Panel **(A)** shows representative averages and standard deviation from three independent assays, data in panels **(B–F)** are averages and standard deviations of data from three biological replicates. Statistical analyses **(A–C)**: unpaired *t*-tests, two-tailed; **(D–F)**: 2-Way ANOVA, Sidaks multiple comparison correction, *p < 0.05, **p < 0.01, ****p < 0.0001.

The mutation was confirmed using PCR, and enzyme assays showed that in crude extracts from the mutant strain, S-oxide reductase activity was reduced by ~80% (Figure 4A). As the Hi2019^Δ*dmsA*^ strain lacked a distinct *in vitro* growth phenotype but showed increased biofilm formation, we then tested biofilm formation in Hi86028NP^Δ*dmsA*^ to confirm a similarity of phenotypes. Under microaerobic conditions, the Hi86028NP^Δ*dmsA*^ strains showed a 5-fold increase in biofilm formation (1-Way ANOVA, Dunnett, *p* < 0.0001) and similar viable cell counts to the wildtype (Figures 4B,C). In addition to oxidizing compounds, reactive nitrogen species are also commonly found at sites of infection (Ulfig and Leichert, 2021), and we therefore tested the susceptibility of the Hi2019 and 86-028NP *dmsA* mutant strains to NO stress, however, both strains behaved the same as the respective wildtype strains (Supplementary Figure S4).

In infection assays using 16HBE14 bronchial epithelial cells, the 86-028NP *dmsA* mutant strain showed a reduction in total cell-associated bacteria of 1.6- and 7.7-fold relative to the wildtype strain at 4 h and 24 h p.i. (2-Way ANOVA, Sidak, *p* = 0.0255 & 0.023), while intracellular bacterial loads were reduced 8-fold at 4 h and 100-fold at 24 h p.i. (2-Way ANOVA, Sidak, *p* = 0.0001 & 0.001) (Figures 4D,E). Planktonic cell numbers showed no statistically significant differences (Figure 4F). These results resemble the phenotype we reported for Hi2019^Δ*dmsA*^ (Dhouib et al., 2021), except that for the 86-028NP Δ*dmsA* strain, the infection phenotype was somewhat more pronounced. In infections of mouse primary macrophages, the 86-028NP Δ*dmsA* strain showed no statistically significant difference in survival compared to the WT strain, which differs from the behavior of the corresponding Hi2019 derivative strain that showed increased survival at 6 h post-infection (Supplementary Figure S2). The slight differences in infection phenotypes may be due to the absence of the MtsZ methionine sulfoxide reductase that provides some functional redundancy in *H. influenzae* 2019.

Together, these experiments demonstrate that the phenotype of the *H. influenzae* 86-028NP Δ*dmsA* strain is similar to what we previously described for the equivalent mutation in Hi2019 (Dhouib et al., 2021), but in the tissue cell infection model, the *H. influenzae* 86-028NP Δ*dmsA* strain showed an increased colonization defect for tissue cell adherent bacteria.

### HiDmsABC is not only able to convert S-oxides but is also highly specific for several biologically relevant N-oxide molecules

**Figure 5:**
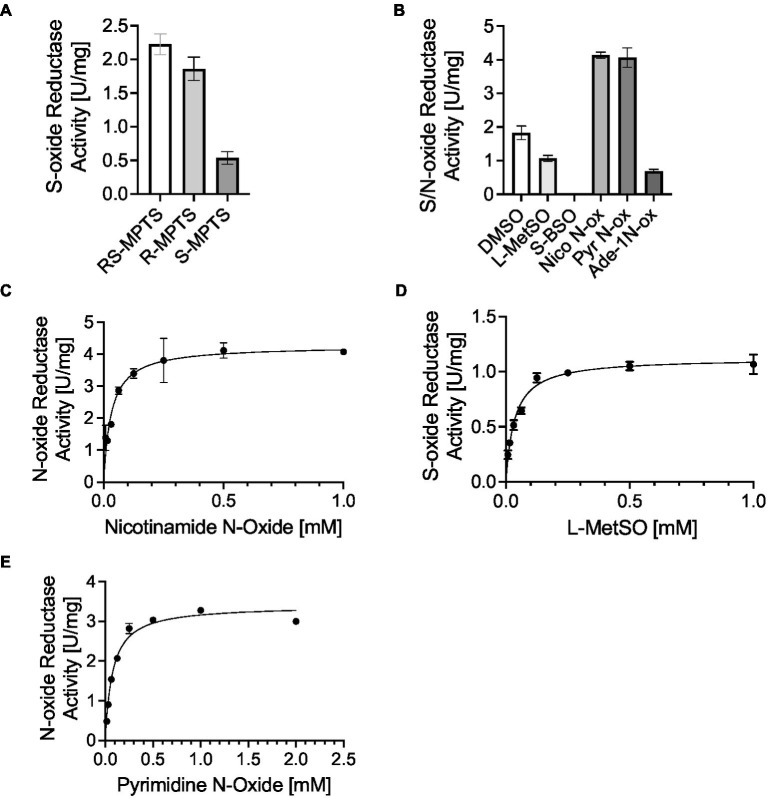
*H. influenzae* DmsABC S- and N-oxide reductase activity. **(A)** Stereospecificity of DmsABC S-oxide reductase activity. **(B)** S-and N-oxide reductase activity in cell extracts of anaerobically grown *H. influenzae* 86-028NP with various substrates. DMSO, dimethyl sulfoxide; L-MetSO, L-methionine (R/S) sulfoxide; S-BSO, S-biotin sulfoxide; Nico-N-ox, Nicotinamide-n-oxide; Pyr-N-ox, pyrimidine-N-oxide; Ade-1 N-ox, Adenine 1-N-oxide. **(C–E)** Substrate dependence of DmsABC activity in cell extracts of anaerobically grown *H. influenzae* 86-028NP with Nicotinamide-n-oxide, L-methionine sulfoxide and pyrimidine N-oxide. All data shown are averages and standard deviation of three independent assays.

Our data so far indicate that DmsABC is important for full virulence of *H. influenzae*, with strong phenotypes observed only during infection. This strongly suggests that the substrate that DmsABC converts is only available during contact with host cells. As the enzyme is produced in the presence of hypochlorite and paraquat-mediated stress, this suggests that the natural substrate could be an oxidatively damaged biomolecule. To better understand the catalytic properties of HiDmsABC, we used cell extracts of the MtsZ negative Hi86-028NP wildtype strain that has high S-oxide reductase activity (Figures 3D,E). Initial experiments used the artificial substrate Methyl-p-tolyl-sulfoxide (MPTS) to determine the HiDmsABC stereospecificity. Activity with racemic RS-MPTS was 2.23 U/mg, while R- and S-MPTS resulted in 1.86 U/mg and 0.54 U/mg of activity (Figure 5A). This confirmed that, like its *E. coli* counterpart (SimalaGrant and Weiner, 1996), HiDmsABC is specific for R-sulfoxides and thus has the opposite stereospecificity to HiMtsZ, which is an S-sulfoxide-specific enzyme (Dhouib et al., 2016). We then tested HiDmsABC activity with different S- and N-oxides that could occur in the human respiratory tract and DMSO as a control substrate. HiDmsABC easily converted L-methionine S/R sulfoxide, nicotinamide-N-oxide, pyrimidine-N-oxide and can also catalyze reduction of adenine-N-1-oxide, while S-Biotin Sulfoxide was not converted and was not investigated further (Figure 5B). As expected, due to the higher reactivity of N-oxides compared to S-oxides, DmsABC activities were highest with nicotinamide-N-oxide and pyrimidine-N-oxide (~4 U/mg), but interestingly the *K*_M_-values for both substrates were in the low micromolar ranges with 32 ± 13 μM, and 76 ± 17 μM, and were, in fact, in a similar range as the *K*_M_-value for L-methionine S/R sulfoxide (35 ± 9 μM) (Figures 5C–E). These catalytic parameters make all three compounds excellent candidates as natural DmsABC substrates. In contrast, adenine-N-1 oxide had a *K*_M_-value of 793 ± 138 μM, making it an unlikely natural substrate for DmsABC. Effective conversion of nicotinamide- and pyrimidine N-oxide by DmsABC has not previously been described but is an important discovery as *H. influenzae* is an auxotroph for both pyrimidine and NAD, for which nicotinamide is a precursor. With the exception of adenine-N-1-oxide, all *K*_M_-values are not only within a concentration range that is highly likely to be physiologically relevant, but the *K*_M_-value for L-methionine sulfoxide is also comparable to the value of 90 μM that has been previously reported for DmsABC from *E. coli* (SimalaGrant and Weiner, 1996).

### DmsABC is an essential part of a novel, conserved defense against sulfoxide stress in *Haemophilus influenzae*

In addition to DmsABC, *H. influenzae* strains contain two other periplasmic sulfoxide reductases, the MtsZ methionine sulfoxide reductase and the MsrAB peptide methionine sulfoxide reductase (Dhouib et al., 2016; Nasreen et al., 2020, 2022). The three enzymes reduce either protein-bound methionine sulfoxide (MsrAB) or free S-/N-oxides (MtsZ, DmsABC), and their expression is upregulated when the bacteria are exposed to hypochlorite (Dhouib et al., 2016; Nasreen et al., 2020). We hypothesized that together, these three enzymes form a new system for protection of *H. influenzae* from oxidative stress, and to fully define the roles of these three methionine sulfoxide reductases in this process, we constructed *H. influenzae* 2019 double mutant strains that each lack two of the three S-oxide reductases.

S-oxide reductase activity assays were used to confirm the contribution of DmsA and MtsZ to the reduction of small-molecule S-oxides in the double mutant strains. As expected, double mutants that lacked MtsZ showed only very low levels of S-oxide reductase activity with both L-methionine sulfoxide and DMSO (Supplementary Figures S5A,B), with lowest activities observed for the Δ*dmsA*Δ*mtsZ* strain. Unexpectedly, the Δ*dmsA*Δ*msrAB* strain in which MtsZ is the only sulfoxide reductase showed about 35% reduced activity, which is about 4x more than expected based on the activity levels (7% of WT) detected in the Δ*msrAB*Δ*mtsZ* strain and could indicate some synergistic effects.

**Figure 6:**
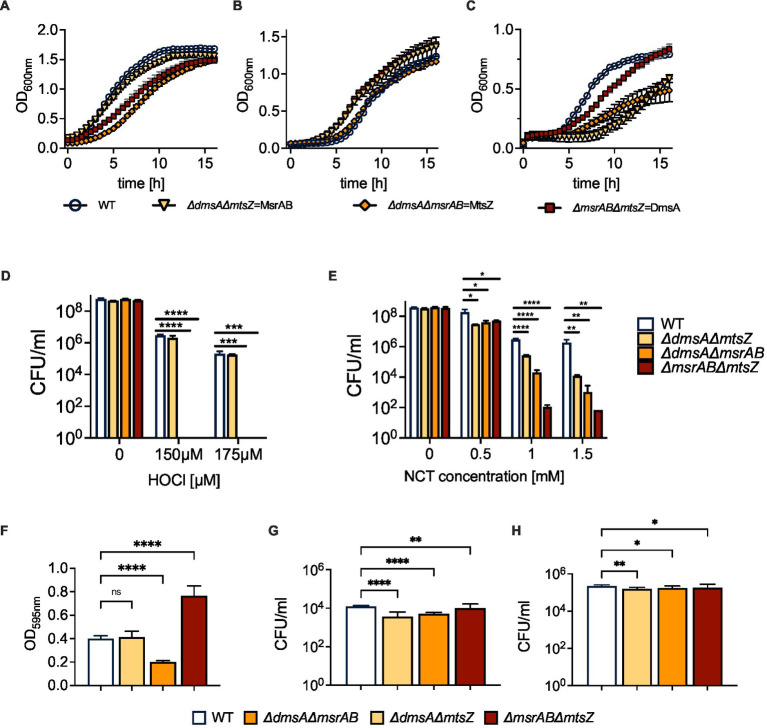
Properties of *H. influenzae* S-oxide reductase double mutant strains. **(A–C)** Growth of *H. influenzae* 2019 S-oxide reductase double mutants under aerobic, microaerobic and anaerobic conditions on Chemically Defined Medium (CDM). **(D,E)** Susceptibility of Hi2019 S-oxide reductase double mutants to exposure to HOCl (D) or N-Chlorotaurine **(E)** for 60 min. **(F)** Biofilm formation in Hi2019 S-oxide reductase double mutants in 96-well plates under microaerobic conditions. **(G,H)** Intracellular bacterial CFU/ml following 4 h **(G)** and 24 h **(H)** infection of 16HBE14 bronchial epithelial tissue cells with Hi2019 S-oxide reductase double mutants. All data shown are averages and standard deviations of at least three biological replicates. Statistical analyses **(D,E,G,H)**: 2-Way ANOVA, Dunnett’s multiple comparison correction, *p < 0.05, **p < 0.01, ****p < 0.0001. **(F)**: 1-Way ANOVA, Dunnett’s multiple comparison correction, ****p < 0.0001.

We then determined growth of the double mutant strains in the presence and absence of oxygen. Growth of all strains was very similar under microaerobic conditions. However, compared to the WT strain, strains carrying a *msrAB* gene knockout (Δ*dmsA*Δ*msrAB;* Δ*msrAB*Δ*mtsZ*) showed reduced growth under aerobic conditions, and interestingly, under anaerobic conditions, strains carrying a *dmsA* gene knockout (Δ*dmsA*Δ*msrAB;* Δ*dmsA*Δ*mtsZ*) showed reduced growth (Figures 6A–C; Supplementary Table S3). For the MsrAB sulfoxide reductase, a similar phenotype was already apparent in the single mutant strain. However, the *dmsA* single mutant strain did not show a reduced growth rate compared to the WT strain (Nasreen et al., 2020; Dhouib et al., 2021).

As all three enzymes have a role in oxidative stress responses, we also determined the sensitivity of the double mutant strains to oxidative stress caused by hydrogen peroxide or the two reactive chlorine species, HOCl or N-Chlorotaurine (NCT). Compared to the WT strain, the double mutants showed a small but statistically significant increase in H_2_O_2_ sensitivity at high concentrations that are unlikely to be physiologically relevant. However, when the double mutant strains were exposed to HOCl, strains that lacked the *msrAB* gene were undetectable following exposure to both 150 and 175 μM HOCl, clearly linking the presence of *msrAB* to *H. influenzae* HOCl resistance (Figure 6D). We then tested resistance of the strains to another reactive chlorine species, N-Chlorotaurine (NCT), expecting to see a similar, but weaker phenotype as NCT is less reactive than HOCl. Following exposure to NCT, strains lacking the *msrAB* gene were again most strongly affected, however, reductions in CFU were more pronounced for the Δ*msrAB*Δ*mtsZ* strain, where a 10^6^-fold decrease in CFU/ml relative to the wildtype strains was observed at concentrations of both 1 mM and 1.5 mM NCT, compared to 10^4^- and 10^5^-fold reductions for Δ*dmsA*Δ*msrAB*. Unexpectedly, attenuation was also observed for the Δ*dmsA*Δ*mtsZ* strain that was unaffected by HOCl exposure but showed a ~ 600- and 10^4^-fold reduction in CFU/ml at 1 mM and 1.5 mM NCT (Figure 6E). These changes in attenuation demonstrate that while MsrAB is the most important enzyme for defense against RCS-induced stress, the two Mo-containing S-oxide reductases, and particularly MtsZ, also contribute to NTHi survival during NCT stress.

### The presence and absence of *Haemophilus influenzae* S-oxide reductases alters biofilm formation profiles

Biofilm formation is a typical bacterial response to cellular stress, and in *H. influenzae,* biofilm formation has been linked to colonization of host cells and tissues. Under microaerobic conditions, two of the three mutant strains showed altered biofilm formation. The Δ*dmsA*Δ*msrAB* mutant strain, in which MtsZ is the only sulfoxide reductase, showed a ~ 50% reduction in biofilm formation relative to the wild type. In contrast, in the Δ*msrAB*Δ*mtsZ* strain, where DmsABC is the only sulfoxide reductase, biofilm formation was increased ~2-fold (Figure 6F). The third mutant strain, Δ*dmsA*Δ*mtsZ*, showed wildtype levels of biofilm formation.

The link between the presence and absence of these Mo-containing S-oxide reductases and biofilm formation is currently unknown but warrants further investigation. It is intriguing that when present as the sole S-/N-oxide reductases, MtsZ and DmsABC appear to have opposing effects on biofilm formation.

### DmsABC is required for successful invasion of tissue cells

To assess the impact of the double mutants on *H. influenzae* virulence, we infected 16HBE14 cultured bronchial epithelial cells with either the WT or one of the mutant strains. At 4 h post-infection, the mutant strains showed modest reductions in total cell numbers of 12% (Δ*dmsA*Δ*mtsZ*), 21% (Δ*dmsA*Δ*msrAB*) and 24% (Δ*msrAB*Δ*mtsZ*) (Figures 6G,H; Supplementary Figure S6). However, clear differences existed for intracellular populations, which were reduced by 60 and 70% for Δ*dmsA*Δ*msrAB* and Δ*dmsA*Δ*mtsZ*, while for the Δ*msrAB*Δ*mtsZ* mutant, only a modest reduction of intracellular bacteria by 20% compared to the WT was observed. By 24 h, CFUs for all strains had increased, and all mutant strains showed ~40% reduction in total cell numbers compared to the WT. Intracellular cell numbers, however, showed the same trends as at 4 h, with the greatest reductions seen for the *dmsA*Δ*mtsZ* mutant (30% reduction), while the Δ*msrAB*Δ*mtsZ* strain was, again, the least affected (20% reduction) (Figures 6G,H; Supplementary Figure S6). Together, the data indicate that MsrAB (Δ*dmsA*Δ*mtsZ* strain) is not required for invasion of tissue cells but may affect extracellular survival of *H. influenzae*. In contrast, the two Mo-containing S-/N-oxide reductases are required for intracellular survival. Of the two enzymes, DmsABC had the greatest impact on tissue cell invasion, while MtsZ contributed to invasion but was less critical than DmsABC, as documented by the reduction of bacterial populations in the Δ*dmsA*Δ*msrAB* strain.

## Discussion

*Haemophilus influenzae* strains are known for their ability to withstand oxidative stress, which is part of their success as host-adapted human pathogens. The bacterial cell envelope is the area where host-produced stressors such as hydrogen peroxide, hypochlorite and others first impact the bacterial cell. While several oxidative stress defense systems have been previously described in *H. influenzae*, the relevant enzymes, such as peroxiredoxin, catalase and superoxide dismutase, are located in the *H. influenzae* cytoplasm (Harrison et al., 2007, 2012, 2015; Pang et al., 2012). Additionally, these known *H. influenzae* oxidative stress responses were mostly identified using exposure of the bacteria to hydrogen peroxide (Harrison et al., 2007; Wong et al., 2007).

**Figure 7:**
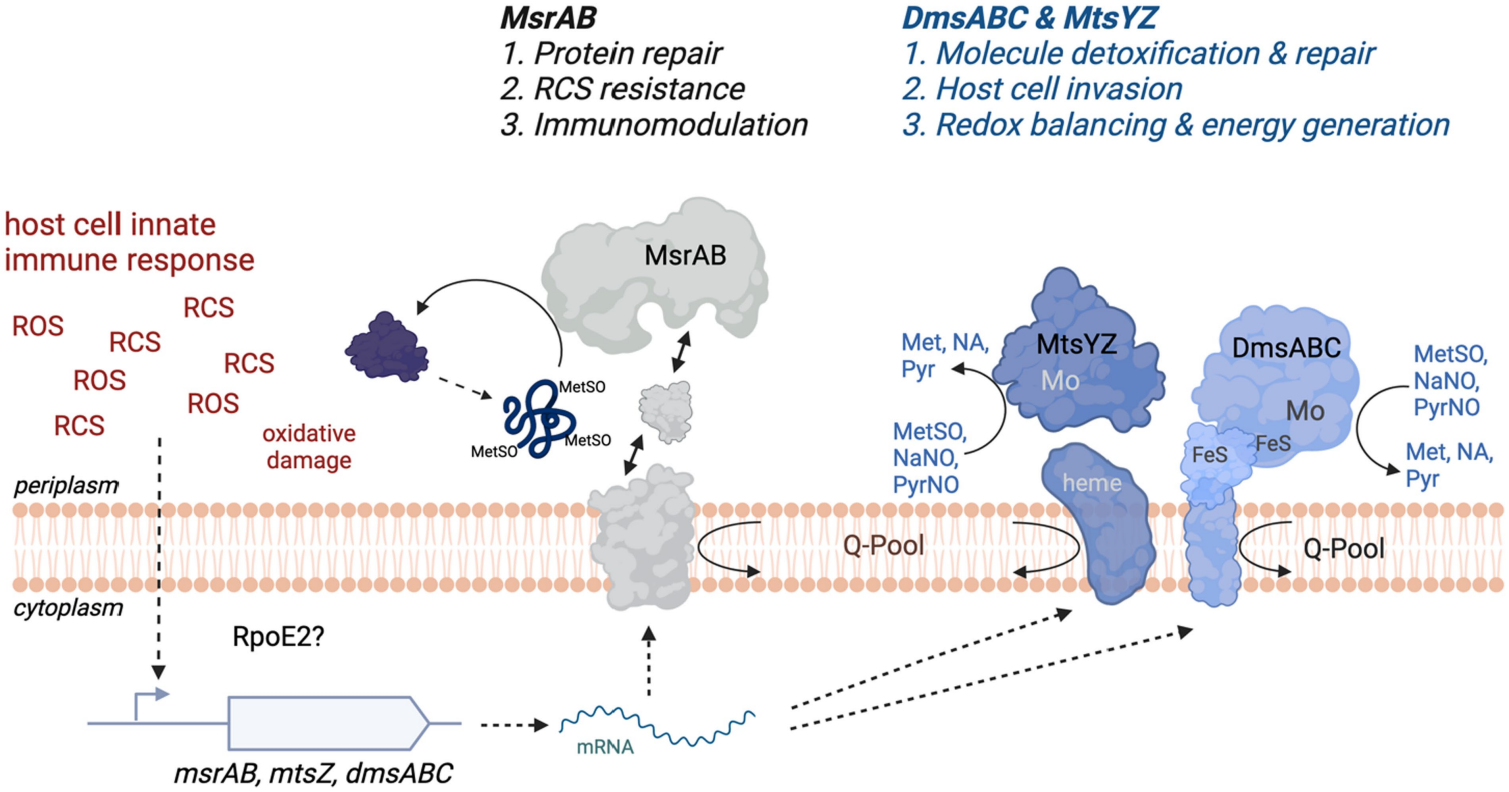
Schematic Representation of the function of the S-/N-oxide damage repair system in *H. influenzae*. FeS, iron sulfur cluster; Met, methionine; MetSO, methionine sulfoxide; Mo, molybdenum active site of MtsZ and DmsABC; NA, nicotinamide; NaNO, Nicotinamide-N-oxide; Pyr, pyrimidine; PyrNO, pyrimidine-N-oxide; RCS, Reactive Chlorine Species such as HOCl or N-Chlorotaurine; ROS, Reactive Oxygen Species such as hydrogen peroxide and superoxide; Q-Pool, cellular quinone pool. Figure created with BioRender.com.

Here, we have characterized a novel, extracellular stress defense system in *H. influenzae* that is composed of two types of sulfoxide reductases, the peptide methionine sulfoxide reductase MsrAB and the two molybdenum-dependent S-oxide reductases DmsABC and MtsZ (Figure 7). Expression of all three enzymes increased in the presence of reactive chlorine species (RCS), but also superoxide-producing compounds such as paraquat. While hypochlorite-induced gene expression had been previously demonstrated for MsrAB and MtsZ, this is the first time that specific inducers of gene expression have been identified for DmsABC. To date, the only regulators that have been shown to modulate expression of DmsABC in bacteria are the Fumarate and Nitrate Reductase regulator (FNR) that controls gene expression in response to anaerobiosis, and the ModE molybdate responsive regulator (McNicholas et al., 1998; Bearson et al., 2002; Harrington et al., 2009). Both of these control gene expression in response to very general conditions that may be frequently encountered by bacteria and specific inducers of *dmsABC* expression had so far not been identified. However, in *E. coli*, nitrate can act as a specific repressor of *dmsABC* expression (Bearson et al., 2002). The induction of *H. influenzae msrAB*, *mtsZ*, and *dmsABC* by reactive oxygen and chlorine species known to be produced by the human immune system documents a novel link between especially the two molybdo-enzymes, MtsZ and DmsABC, and protection from oxidative stress during infection. Traditionally, this type of enzyme has been associated with bacterial energy generation under anaerobic conditions as catalysis uses quinols that mediate electron transfer between respiratory chain complexes as the electron donors (Kappler and Schaefer, 2014; Kappler et al., 2019). At present, only a single hypochlorite-responsive regulator, the RpoE2 extracytoplasmic function—sigma factor, has been identified in *H. influenzae* (Nasreen et al., 2021), and this regulator might mediate the hypochlorite-based induction of *dmsABC* expression.

Although MsrAB, MtsZ, and DmsABC are all induced following exposure of the bacteria to RCS and ROS, the functions of these enzymes in protecting *H. influenzae* from RCS/ROS stress are distinct (Figure 7). Our experiments using S-oxide reductase double mutant strains demonstrate that MsrAB mediates resistance to RCS-killing, likely by repairing oxidative damage to a range of periplasmic and outer membrane proteins that we characterized previously (Nasreen et al., 2020, 2022). Additionally, MsrAB is also involved in host immunomodulatory activities during *H. influenzae* infections (Nasreen et al., 2020) (Figure 7). Conversely, the two Mo-dependent S/N-oxide reductases, MtsZ and DmsABC, appear to be involved in repairing damage to small molecule S- and N-oxides, and there is some functional redundancy (Dhouib et al., 2016, 2021; Struwe et al., 2021). The double mutant studies showed that both enzymes have a similar functional profile during host interactions, where they are required for efficient colonization of the intracellular environment. Interestingly, our single mutant studies also revealed that in mouse infections, the Hi2019 Δ*dmsA* strain was less immunogenic and elicited a reduced cytokine response that led to lower numbers of immune cells detected in BALF samples. The reason for this reduced immunogenicity is currently unclear as DmsABC is located in the periplasmic space and thus should not be surface-exposed. We have, however, previously observed a similar effect in Hi2019 strains lacking a membrane-associated enzyme required for energy generation from lactate (Hosmer et al., 2022), and propose that a possible explanation for the reduced immunogenicity in both of these strains might be a sub-optimal use of the metabolic network and lack of redox balancing in these strains. DmsABC and MtsZ also played a minor role in resistance to stressors such as NCT. *H. influenzae* strains lacking either of these enzymes only exhibited pronounced phenotypes during host colonization. DmsABC mutant strains had a much more pronounced phenotype, indicating that this enzyme is more important for *H. influenzae* survival in contact with host cells. In keeping with the greater importance of DmsABC, MtsZ is absent in about 20% of *H. influenzae* strains, while DmsABC, like MsrAB, is strictly conserved in all strains. Our data show that in *H. influenzae* strains that lack MtsZ, phenotypes generated by a *dmsA* gene knockout are more pronounced (Figure 4), supporting the functional redundancy of the two enzymes.

An interesting, yet so far not fully resolved question is what substrates are converted by the Mo-containing S-oxide reductases during contact with the host where they are required for *H. influenzae* survival. Using a selection of biologically relevant S- and N-oxides that could occur at sites of infection where high levels of oxidizing compounds are present (Ulfig and Leichert, 2021), we have shown here that DmsABC converts not only methionine sulfoxide but also nicotinamide- and pyrimidine-N-oxides with great efficiency and *K*_M_-values that are in the physiological range. This is significant, as nicotinamide is a precursor for NAD, for which *H. influenzae* is an auxotroph, and can be converted to NAD via the actions of DeoD, NadN, SurE and NadR. Similarly, *H. influenzae* strains are pyrimidine auxotrophs and rely on pyrimidine salvage to meet cellular pyrimidine requirements (Tatusov et al., 1996). We had previously established that MtsZ, like DmsABC, is an efficient methionine sulfoxide reductase [9; 25], and kinetic analyses with nicotinamide- and pyrimidine-N-oxide as substrates revealed that MtsZ also converts both of these substrates with high specificity (Nicotinamide-N-oxide: *K*_M:_ 46 ± 8 μM, *k*_cat_: 93 ± 5 s^−1^; Pyrimidine-N-oxide: *K*_M:_ 35 ± 8 μM, *k*_cat_: 33.2 ± 2.7 s^−1^) (Supplementary Figure S7). Despite the similarities in catalytic profiles, an interesting difference between MtsZ and DmsABC is their opposing stereospecificity (Dhouib et al., 2016; Struwe et al., 2021), which, however, does not appear to affect their ability to fulfill similar physiological roles. We propose that this could be due to the non-chiral nature of the reaction products produced by these enzymes from chiral precursors such as S-oxides, where the final product is the same regardless of the configuration of the substrate molecule.

Lastly, our discovery of the physiological role of *H. influenzae* DmsABC as a major enzyme in host interactions rather than an enzyme mediating anaerobic respiration with DMSO, suggests that the latter role may be an artifact and have little relevance under physiological conditions for *H. influenzae*, i.e., during contact with host respiratory epithelia. Based on our results, we propose that homologs of DmsABC that exist in many pathogenic and commensal bacteria, including in *E. coli,* may, in fact, in most cases, underpin successful host colonization rather than anaerobic respiration with DMSO. As DMSO is a molecule that may occur in some aquatic environments, but generally does not occur in significant amounts in vertebrate bodies, the physiological role of DmsABC in *E. coli* where DMSO respiration is the accepted role should be reinvestigated (Sambasivarao and Weiner, 1991; Kappler and Schaefer, 2014). Our proposal is supported by studies in *Actinobacillus pleuropneumoniae* and *Salmonella enterica* that also identified DmsABC and homologous enzymes as essential for virulence (Baltes et al., 2003; Cruz et al., 2023). If the universality of this role and the interplay between peptide methionine sulfoxide reductases such as MsrAB and Mo-containing S-oxide reductases could be demonstrated, this would make these two classes of enzymes attractive, bacteria-specific drug targets.

## Data availability statement

The original contributions presented in the study are included in the article/Supplementary material, further inquiries can be directed to the corresponding author.

## Ethics statement

The studies involving humans were approved by University of Queensland Ethics Committee (Approvals #2017000520, 2021/HE001644, 2014/HE000067). The studies were conducted in accordance with the local legislation and institutional requirements. The participants provided their written informed consent to participate in this study. The animal study was approved by the Animal Care and Ethics Committees of QIMR Berghofer and the University of Queensland (QIMR/050/19). The study was conducted in accordance with the local legislation and institutional requirements.

## Author contributions

MN: Writing – review & editing, Writing – original draft, Visualization, Methodology, Investigation, Formal Analysis. DE: Writing – review & editing, Visualization, Methodology, Investigation. JH: Writing – review & editing, Visualization, Methodology, Investigation, Formal Analysis. A-TE: Writing – review & editing, Supervision, Project administration, Methodology, Investigation. EF: Writing – review & editing, Supervision, Methodology, Investigation, Formal Analysis. PS: Writing – review & editing, Supervision, Methodology. AM: Writing – review & editing, Supervision, Project administration, Funding acquisition. UK: Writing – review & editing, Writing – original draft, Visualization, Supervision, Project administration, Funding acquisition, Conceptualization.
