# Supplementary material for: The DmsABC S-oxide reductase is an essential component of a novel, hypochlorite-inducible system of extracellular stress defense in Haemophilus influenzae
Source: Front Microbiol. 2024 Apr 4;15:1359513. doi: 10.3389/fmicb.2024.1359513 (PMC11024254; doi:10.3389/fmicb.2024.1359513)
Supplement: Supplementary file 1 [file Data_Sheet_1.DOCX]

The DmsABC S-oxide reductase is an essential component of a new system of extracellular stress defence in *Haemophilus influenzae*

SUPPLEMENTARY DATA & FIGURES

Marufa Nasreen^1^, Daniel Ellis^1^, Jennifer Hosmer^1^, Ama-Tawiah Essilfie^2^, Emmanuelle Fantino^3^, Peter Sly^3^, Alastair G. McEwan^1^, Ulrike Kappler^1^

^1^School of Chemistry and Molecular Biosciences, Australian Infectious Diseases Research Centre, The University of Queensland, St. Lucia, Qld 4072, Australia

^2^QIMR Berghofer Medical Research Institute, 300 Herston Road, Herston QLD 4006, Australia

^3^Child Health Research Centre, 62 Graham St, South Brisbane QLD 410, Australia

**Figure S1:** Comparison of mouse lung infections with Hi2019^WT^ and Hi2019^Δ^*^dmsA^*. **Panel A:** Bacteria colony forming unit (CFU) recovered from mouse Broncho-Alveolar Lavage Fluid (BALF). **Panels B-D:** relative expression of HIF1α, TGFB and BIRC3 in mouse lung tissue during infection with NTHi strains. qPCR data was normalized against expression of ACTB, cDNA was generated using random hexamers. **Panels E:** TNFα levels in mouse BALF determined by ELISA. **Panels F-H:** Neutrophil, macrophage and lymphocyte cell counts (Giemsa stain) in mouse BALF.

Statistical analyses: Panel A: multiple un-paired t-tests, **** p<0.0001; Other Panels: 2-Way ANOVA, Sidaks multiple comparison correction, * p<0.05, ** p<0.01, **** p<0.0001

**Figure S2:** Intracellular survival of Hi2019 (Panel A) and 86-028NP (Panel B) WT and Δ*dmsA* strains in bone marrow derived murine macrophages. Following infection (1h), treatment with polymyxin and use of a polymyxin maintenance dose was used to ensure that only intracellular bacteria were present in the assay.

Statistical analyses for intracellular CFU/mL: 2-Way ANOVA, Sidaks’ multiple comparison correction, **** p<0.0001.

**Figure S3:** Transepithelial resistance (TEER) of uninfected NHNE compared to NHNE infected with an equal mixture of Hi2019^WT^ and Hi2019^Δ^*^dmsA^*.

**Figure S4:** Nitrosative stress resistance in *H. influenzae* wild-type and Δ*dmsA* strains. Experiments were carried out as described in the Methods section, data shown are averages of three technical replicates, the experiment was repeated twice on different days and a single, a representative dataset is shown.

Statistical analysis by 2-Way ANOVA with Tukey’s multi-comparison correction was carried out and revealed that comparisons between strains at a given treatment concentration returned non-significant changes.

**Figure S5:** S-oxide reductase activity in *H influenzae* sulfoxide reductase double mutant strains. Substrates used: Panel A: DMSO, Panel B: L-methionine (S/R) sulfoxide. Data shown are averages and standard deviation of at least three independent enzyme assays.

Statistical testing used 1-Way-ANOVA with Dunnet’s multicomparison correction. **** p<0.0001

**Figure S6:** Infection of 16HBE14 human bronchial epithelial tissue cells with *H. influenzae* Hi2019 S-oxide reductase double mutant strains for 4h or 24 h. Data shown are averages and standard deviation of three biological replicates.

Statistical analyses: 2-Way ANOVA, Sidaks’ multi-comparison correction, * p<0.05, **p<0.01, **** p<0.0001

**Figure S7:** Activity of purified *H. influenzae* MtsZ with Nicotinamide N-Oxide and Pyrimidine -N-oxide. Kinetic parameters: Nicotinamide-N-oxide: *K*_M:_ 46±8 μM, *k*_cat_: 93±5 s^-1^; Pyrimidine-N-oxide: *K*_M:_ 35±8 μM, *k*_cat_: 33.2±2.7 s^-1^

**Table S1:** Bacterial strains and plasmids used in this study.

| **Strain** | **Description** | **Source/Ref** |
| --- | --- | --- |
| *Escherichia coli* DH5α | F– φ80lacZΔM15 Δ(lacZYA-  argF)U169 recA1 endA1  hsdR17(rK–, mK+) phoA supE44  λ– thi-1 gyrA96 relA1, cloning  strain | Life Technologies |
| *Haemophilus influenzae* 2019 | Clinical isolate from a chronic  obstructive pulmonary disease  patient.  Sequence type 321 | (1) |
| *Haemophilus influenzae* 86-  028NP | Clinical isolate from a patient  with otitis media.  Sequence type 33 | (2) |
| Hi3 | Clinical isolate, UQ collection | This study |
| Hi2019*^ΔdmsA^* | Hi2019^WT^ with *dmsA* gene  disrupted by the insertion of a  kanamycin antibiotic resistance  cassette  (*dmsA*::*kan*) | (3) |
| Hi2019*^ΔmsrAB^* | Hi2019^WT^ with *msrAB* gene  disrupted by the insertion of a  kanamycin antibiotic resistance  cassette  (*msrAB::kan*) | (4) |
| Hi2019*^ΔmtsZ^* | Hi2019^WT^ with *mtsZ* gene  disrupted by the insertion of a  kanamycin antibiotic resistance  cassette  (*mtsZ::kan*) | (5) |
| 86028*^ΔdmsA^* | 86028^WT^ with *dmsA* gene  disrupted by the insertion of a  kanamycin antibiotic resistance  cassette  (*dmsA*:*:kan*) | This study |
| Hi2019*^ΔdmsAΔmtsZ^* | Hi2019^WT^ with *dmsA* gene  disrupted by the insertion of a  kanamycin antibiotic resistance  cassette and with *mtsZ* gene disrupted by the insertion of a tetracycline antibiotic resistance cassette  (*dmsA*::*kan* and *mtsZ*:*:tet*) | This study |
| Hi2019*^ΔmsrABΔmtsZ^* | Hi2019^WT^ with *msrAB* gene  disrupted by the insertion of a  kanamycin antibiotic resistance  cassette and with *mtsZ* gene disrupted by the insertion of a tetracycline antibiotic resistance cassette  (*msrAB*::*kan* and *mtsZ*::*tet*) | This study |
| Hi2019*^ΔdmsAΔmsrAB^* | Hi2019^WT^ with *dmsA* gene  disrupted by the insertion of a  kanamycin antibiotic resistance  cassette and with *msrAB* gene disrupted by the insertion of a tetracycline antibiotic resistance cassette  (*dmsA::kan* and *msrAB*::*tet*) | This study |
| *Actinobacillus pleuropneumoniae* (strain 4074) | Clinical isolate from a swine  with respiratory disease | ATCC |
| **Plasmid** | **Description** | **References or Source** |
| pUC4K | Cloning vector used to isolate  the kanamycin resistance  cassette. Vector also contains  ampicillin resistance cassette | (6) |
| pRK415 | Cloning vector used to isolate  the tetracycline resistance  cassette. | (7) |
| pGEM-T Easy | Cloning vector | Promega |
| pBluescript II SK+ | Cloning vector | Stratagene |
| pGEM-Hi*dmsA* | pGEM-T Easy derivative  containing a 1000bp DNA  fragment carrying the *dmsA*  gene and flanking regions | (3) |
| pGEM-Hi*dmsA::kan* | pGEM-Hi*dmsA* with the dmsA  gene disrupted by a kanamycin  antibiotic resistance cassette | (3) |
| pGEM-Hi*mtsZ* | pGEM-T Easy derivative  containing a 1000bp DNA  fragment carrying the *mtsZ*  gene and flanking regions | (5) |
| pGEM-Hi*mtsZ::kan* | pGEM-Hi*mtsZ* with the *mtsZ*  gene disrupted by a kanamycin  antibiotic resistance cassette | (5) |
| pBlue-Hi*msrAB* | pBluescript derivative  containing a 1000bp DNA  fragment carrying the *msrAB* gene and flanking regions | (4) |
| pBlue-Hi*msrAB::kan* | pBlue-Hi-msrAB with the *msrAB* gene disrupted by a kanamycin antibiotic resistance cassette | (4) |
| pGEM-Hi*mtsZ::tet* | pGEM-Hi*mtsZ* with the *mtsZ*  gene disrupted by a tetracycline  antibiotic resistance cassette | This study |
| pBlu-Hi*msrAB::tet* | pGEM-Hi*msrAB* with the *msrAB* gene disrupted by a tetracycline  antibiotic resistance cassette | This study |

**Table S2: Oligonucleotide primers used in this study.**

| **Primer for pGEM-Hi*dmsA*::kan** | |
| --- | --- |
| HI_dmsA F | CTACAAACGTTCCACTTGAAC |
| HI_dmsA R | ATGAGTAACTTTAATCAAATAAGT |
| pUC4K_PCR F | GTTGGGTAACGCCAGGGTTTTCC |
| pUC4K_PCR_R | TCCGGCTCGTATGTTGTGTGGAA |
| **Primer for pGEM-Hi*mtsZ:*:tet** | |
| torZ_extF | TTA CGC CAC CTG TTT AGG |
| torZ_intR | AAA AGG ATC CGG GGC AAA AAC ATG GTT G |
| torZ_intF | AAA AGG ATC CCG CTT TGC CTG ATG GAC T |
| torZ_extR | ATG AAA AAG AAT AAC GTA AA |
| pRKtetAR_F_Bam | AAAAGGATCCACGCTAGGGCAGGGCATGAAA |
| pRKtetAR_R_Bam | AAAAGGATCCGTCCTGCTCGTGATCGGGA |
| **Primer for pBlu-Hi*msrAB*:tet** | |
| Hi_msrA_XbaI_P1 F | AAAATCTAGATGCAAAAGCGTTTAGGCTGAATGC |
| HI_msrA_BamHI_B1_R | AAAAGGATCCACTCGCCCAGCTTCAAACCAAATA |
| HI_msrA_BamHI_B2_F | AAAAGGATCCGCTTGTCCGATCACCGCTTTATCT |
| HI_msrA_Pst_P4_R | AAAACTGCAGGATGTGGGCGTTAAGGCTGGTTTA |
| **Primer for RT-PCR** | |
| HI_QP0_dmsA_R | CGAACCTGATGATCAAGATTATATG |
| HI_QP0_dmsA_R | AGTAAACTGTGGTAGCCGTTG |
| HI_QP0_gyrA_F | TTGGGCGTGCATTACCTGACGTT |
| HI_QP0_gyrA_R | CCCACAACACGCGCTGATTTTAC |
| Ap Qp gyrA F | AACCGGATCGATATTGGCTAACGC |
| Ap Qp gyrA R | TTCGTCAAACACCGCCGTGAA |
| Ap Qp dmsA F | CCGATTTTAACCGGCAATGTAGGG |
| Ap Qp dmsA R | TGGGATACTTGCTTGCACCGGG |
| Mm-QP-ACTB-F | CTGCGTCTGGACCTGGC |
| Mm-QP-ACTB-R | CTTCTCTTTGATGTCACGCACGAT |
| Mm-QP-BIRC3-F | CTGTGTCAGAAAGGAGTCTGGCT |
| Mm-QP-BIRC3-R | CCATGGGACTGTCCCCTTG |
| Mm-QP-Hif1alpha-F | GCTGGCTCCCTATATCCCAATG |
| Mm-QP-Hif1alpha-R | TGCTGGAACCCAGTAACTGTGC |
| Mm-QP-IL1beta-F | GCTTCAAATCTCGCAGCAGC |
| Mm-QP-IL1beta-R | TCCTCATCCTGGAAGGTCCAC |
| Mm-QP-TNFalpha-F | TGAGCACTGAAAGCATGATCCG |
| Mm-QP-TNFalpha-R | CGATCAGGAAGGAGAAGAGGCTG |
| Mm-QP-IL6-F | GACTTCCATCCAGTTGCCTT |
| Mm-QP-IL6-R | GGTATAGACAGGTCTGTTGG |
| Mm-QP-TGFβ-F | AGAGAAGAACTGCTGTGTGCG |
| Mm-QP-TGFβ-R | ATATAGGGGCAGGGTCCCAG |

**Table S3:**  Growth rates of Hi2019 wildtype and S-/N-oxide reductase double mutant strains under aerobic, microaerobic and anaerobic conditions.

|  | *Growth Rates [h^-1^]* | | |
| --- | --- | --- | --- |
| *Hi2019 strains* | *Aerobic* | *Microaerobic* | *Anaerobic* |
| wildtype | 0.480±0.098 | 0.549±0.045 | 0.397±0.073 |
| Δ*dmsA*Δ*msrAB* | 0.350±0.064 | 0.417±0.050 | 0.253±0.018 |
| Δ*dmsA*Δ*mtsZ* | 0.410±0.099 | 0.448±0.070 | 0.288±0.071 |
| Δ*msrAB*Δ*mtsZ* | 0.349±0.033 | 0.462±0.123 | 0.255±0.025 |

**References:**

1. Campagnari, A. A., Gupta, M., Dudas, K., Murphy, T., and Apicella, M. (**1987**) Antigenic diversity of lipooligosaccharides of nontypable *Haemophilus influenzae*. *Infect. Immun.* **55**, 882-887

2. Harrison, A., Dyer, D. W., Gillaspy, A., Ray, W. C., Mungur, R., Carson, M. B., Zhong, H., Gipson, J., Gipson, M., and Johnson, L. S. (2005) Genomic sequence of an otitis media isolate of nontypeable *Haemophilus influenzae*: comparative study with *H. influenzae* serotype d, strain KW20. *J. Bacteriol.* **187**, 4627-4636

3. Dhouib, R., Nasreen, M., Othman, D., Ellis, D., Lee, S., Essilfie, A. T., Hansbro, P. M., McEwan, A. G., and Kappler, U. (2021) The DmsABC sulfoxide reductase supports virulence in non-typeable *Haemophilus influenzae*. *Front. Microbiol.* **12**, 686833

4. Nasreen, M., Dhouib, R., Hosmer, J., Wijesinghe, H. G. S., Fletcher, A., Mahawar, M., Essilfie, A.-T., Blackall, P. J., McEwan, A. G., and Kappler, U. (2020) Peptide methionine sulfoxide reductase from *Haemophilus influenzae* is required for protection against HOCl and affects the host response to infection. *ACS Infect. Dis.* **6**, 1928-1939

5. Dhouib, R., Othman, D. S. M. P., Lin, V., Lai, X. J., Wijesinghe, H. G., Essilfie, A.-T., Davis, A., Nasreen, M., Bernhardt, P. V., and Hansbro, P. M. (2016) A novel, molybdenum-containing methionine sulfoxide reductase supports survival of *Haemophilus influenzae* in an in vivo model of infection. *Front. Microbiol.* **7**, 1743

6. Vieira, J., and Messing, J. (1982) The pUC plasmids, an M13mp7-derived system for insertion mutagenesis and sequencing with synthetic universal primers. *Gene* **19**, 259-268

7. Keen, N. T., Tamaki, S., Kobayashi, D., and Trollinger, D. (1988) Improved broad-host-range plasmids for DNA cloning in gram-negative bacteria. *Gene* **70**, 191-197

**Table S2:** Oligonucleotide primers used in this study.

| **Primer for pGEM-Hi*dmsA*::kan construction** | |
| --- | --- |
| HI_dmsA F | CTACAAACGTTCCACTTGAAC |
| HI_dmsA R | ATGAGTAACTTTAATCAAATAAGT |
| pUC4K_PCR F | GTTGGGTAACGCCAGGGTTTTCC |
| pUC4K_PCR_R | TCCGGCTCGTATGTTGTGTGGAA |
| **Primer for pGEM-Hi*mtsZ:*:tet construction** | |
| torZ_extF | TTA CGC CAC CTG TTT AGG |
| torZ_intR | AAA AGG ATC CGG GGC AAA AAC ATG GTT G |
| torZ_intF | AAA AGG ATC CCG CTT TGC CTG ATG GAC T |
| torZ_extR | ATG AAA AAG AAT AAC GTA AA |
| pRKtetAR_F_Bam | AAAAGGATCCACGCTAGGGCAGGGCATGAAA |
| pRKtetAR_R_Bam | AAAAGGATCCGTCCTGCTCGTGATCGGGA |
| **Primer for pBlu-Hi*msrAB*:tet construction** | |
| Hi_msrA_XbaI_P1 F | AAAATCTAGATGCAAAAGCGTTTAGGCTGAATGC |
| HI_msrA_BamHI_B1_R | AAAAGGATCCACTCGCCCAGCTTCAAACCAAATA |
| HI_msrA_BamHI_B2_F | AAAAGGATCCGCTTGTCCGATCACCGCTTTATCT |
| HI_msrA_Pst_P4_R | AAAACTGCAGGATGTGGGCGTTAAGGCTGGTTTA |
| **Primer for qRT-PCR** | |
| HI_QP0_dmsA_R | CGAACCTGATGATCAAGATTATATG |
| HI_QP0_dmsA_R | AGTAAACTGTGGTAGCCGTTG |
| HI_QP0_gyrA_F | TTGGGCGTGCATTACCTGACGTT |
| HI_QP0_gyrA_R | CCCACAACACGCGCTGATTTTAC |
| Ap Qp gyrA F | AACCGGATCGATATTGGCTAACGC |
| Ap Qp gyrA R | TTCGTCAAACACCGCCGTGAA |
| Ap Qp dmsA F | CCGATTTTAACCGGCAATGTAGGG |
| Ap Qp dmsA R | TGGGATACTTGCTTGCACCGGG |
| Mm-QP-ACTB-F | CTGCGTCTGGACCTGGC |
| Mm-QP-ACTB-R | CTTCTCTTTGATGTCACGCACGAT |
| Mm-QP-BIRC3-F | CTGTGTCAGAAAGGAGTCTGGCT |
| Mm-QP-BIRC3-R | CCATGGGACTGTCCCCTTG |
| Mm-QP-Hif1alpha-F | GCTGGCTCCCTATATCCCAATG |
| Mm-QP-Hif1alpha-R | TGCTGGAACCCAGTAACTGTGC |
| Mm-QP-IL1beta-F | GCTTCAAATCTCGCAGCAGC |
| Mm-QP-IL1beta-R | TCCTCATCCTGGAAGGTCCAC |
| Mm-QP-TNFalpha-F | TGAGCACTGAAAGCATGATCCG |
| Mm-QP-TNFalpha-R | CGATCAGGAAGGAGAAGAGGCTG |
| Mm-QP-IL6-F | GACTTCCATCCAGTTGCCTT |
| Mm-QP-IL6-R | GGTATAGACAGGTCTGTTGG |
| Mm-QP-TGFβ-F | AGAGAAGAACTGCTGTGTGCG |
| Mm-QP-TGFβ-R | ATATAGGGGCAGGGTCCCAG |

**Table S3:** Growth rates of S-/N-oxide reductase double mutants Growth rates were derived from data collected on three biological replicates (see Figure 6, panels A-C).
